# The SIRT1-HMGB1 axis: Therapeutic potential to ameliorate inflammatory responses and tumor occurrence

**DOI:** 10.3389/fcell.2022.986511

**Published:** 2022-08-19

**Authors:** Lanyi Wei, Wenrui Zhang, Yueyang Li, Jinghui Zhai

**Affiliations:** ^1^ Department of Clinical Pharmacy, The First Hospital of Jilin University, Changchun, Jilin, China; ^2^ Department of Pharmacy, Shanghai Jiao Tong University Affiliated Sixth People’s Hospital, Shanghai, China

**Keywords:** sirtuin 1, high mobility group box 1, inflammation, tumor, neuroinflammation, hepatocyte inflammation

## Abstract

Inflammation is a common complication of many chronic diseases. It includes inflammation of the parenchyma and vascular systems. Sirtuin 1 (SIRT1) is a nicotinamide adenine dinucleotide (NAD)-dependent histone deacetylase, which can directly participate in the suppression of inflammation. It can also regulate the activity of other proteins. Among them, high mobility group box 1 (HMGB1) signaling can be inhibited by deacetylating four lysine residues (55, 88, 90, and 177) in quiescent endothelial cells. HMGB1 is a ubiquitous nuclear protein, once translocated outside the cell, which can interact with various target cell receptors including the receptor for advanced glycation end-products (RAGE), toll-like receptor (TLR) 2, and TLR4 and stimulates the release of pro-inflammatory cyto-/chemokines. And SIRT1 has been reported to inhibit the activity of HMGB1. Both are related to the occurrence and development of inflammation and associated diseases but show an antagonistic relationship in controlling inflammation. Therefore, in this review, we introduce how this signaling axis regulates the emergence of inflammation-related responses and tumor occurrence, providing a new experimental perspective for future inflammation research. In addition, it explores diverse upstream regulators and some natural/synthetic activators of SIRT1 as a possible treatment for inflammatory responses and tumor occurrence which may encourage the development of new anti-inflammatory drugs. Meanwhile, this review also introduces the potential molecular mechanism of the SIRT1-HMGB1 pathway to improve inflammation, suggesting that SIRT1 and HMGB1 proteins may be potential targets for treating inflammation.

## 1 Introduction

Inflammation is an innate host defense mechanism to harmful stimuli and conditions, such as infection and tissue injury, and is an adaptive response that acts to reestablish homeostasis (Medzhitov, 2008). It involves the acute, chronic and resolution phases, which can respectively figure out the injury and initiate the healing process, lead to tissue damage and fibrosis and protect host tissue from chronic or excessive inflammation (Medzhitov, 2008). Acute inflammation mediates innate and humoral immunity, which is the body’s initial protective response, while chronic inflammation leads to the pathology of blood vessels and tissues and is related to various degenerative diseases including arthritis, atherosclerosis, autoimmune diseases, diabetes and cancer (Germolec et al., 2018; Ahmad et al., 2019; Panigrahy et al., 2021). At the cellular level, acute inflammation occurs when innate immune cells sense infectious elements or tissue damage, leading primarily to the recruitment and activation of neutrophils, while chronic inflammation is characterized by a variety of cell types such as neutrophils, monocytes, macrophages, and other immune cells (Pellico et al., 2017). Chronic inflammation is associated with the expression of chemokines, cytokines, and adhesion molecules, which in turn form positive feedback loops that enhance chronic inflammation (Zhang et al., 2018). Therefore, finding appropriate means to control chronic inflammation has always been a hot topic in current research. To reduce chronic inflammation and thus treat diseases caused or exacerbated by this process, a comprehensive understanding of the pathways and molecules involved in inflammation may provide essential information for innovative therapeutic targets.

**FIGURE 1:**
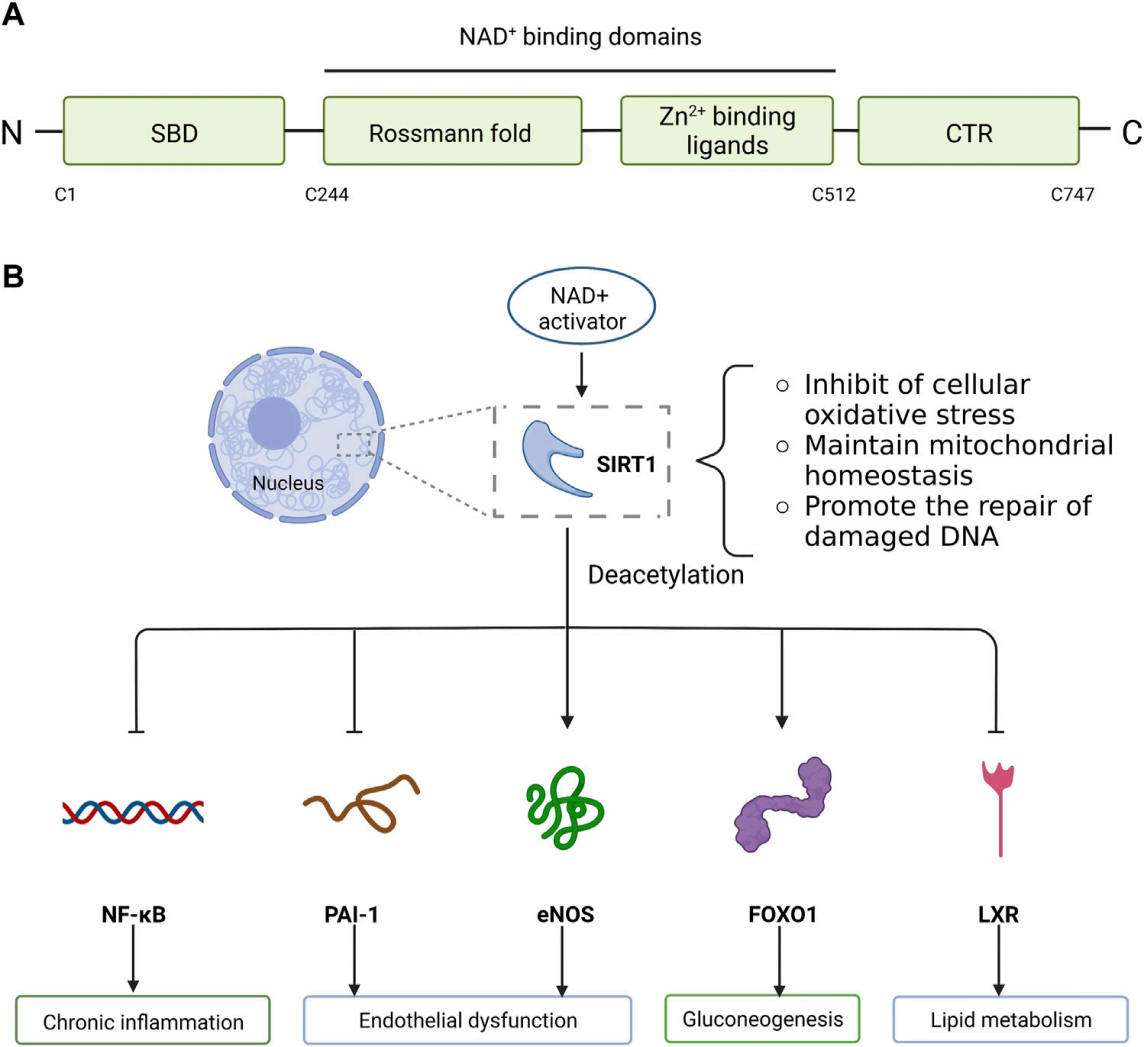
Molecular structure and biological function of SIRT1. **(A)** SIRT1 is composed of N-terminal, C-terminal and NAD^+^ dependent catalytic core region. The catalytic core region (C244-C512) is folded into two subdomains: Zn^2+^ binding ligands and Rossmann fold conformation. N-terminal contains SBD and C-terminal contains CTR. **(B)** SIRT1, mainly located in the nucleus, deacetylates related proteins and reduces cell apoptosis by inhibiting cellular oxidative stress, maintaining mitochondrial metabolic homeostasis, and promoting the repair of damaged DNA. SIRT1 activity is dependent on NAD^+^. The activation of SIRT1 is facilitated by increasing NAD^+^ levels at the cellular level, which can lead to deacetylation and modulated expression of many downstream targets. SIRT1 targets a variety of substrates and performs different functions. SIRT1 deacetylates inflammation-related transcription factor NF-κB, which attenuates NF-κB driven inflammation. In addition, SIRT1 protects endothelial cells against replicative senescence by deacetylating eNOS and downregulating PAI-1 expression. In the liver, SIRT1 deacetylates and activates the transcription factor FOXO1 to stimulate gluconeogenesis. Similarly, SIRT1 regulates lipid metabolism by modulating LXR *via* deacetylation of this molecular receptor. SIRT1, Sirtuin1; SBD, sirtuins-activating compounds binding domain; CTR, C-terminal regulatory segment; NAD^+^, nicotinamide adenine dinucleotide; NF-κB, nuclear factor kappa B; eNOS, endothelial nitric oxide synthase; PAI-1, plasminogen activator inhibitor 1; FOXO1, forkhead box O 1; LXR, liver X-receptor.

Some reports have suggested a link between sirtuin 1 (SIRT1) and high mobility group box 1 (HMGB1) regulation on inflammation (Zhang et al., 2020; Shih et al., 2021). SIRT1 is a nicotinamide adenine dinucleotide (NAD)-dependent histone deacetylase, as a significant regulator of the transcriptional networks that adjust metabolism and stress responses, having a pivotal connection with human being health (Kemper et al., 2013). (Figure 1) HMGB1 is a transcription factor deacetylated by SIRT1. Deacetylated HMGB1 was restricted to translocation out of the nucleus (Zainal et al., 2017). HMGB1 is involved in various pathological and physiological processes. Under normal physiological conditions, nuclear protein HMGB1 is expressed in virtually all eukaryotic cells (Cottone et al., 2016) that protects the cell from apoptotic cell death, which is an integral part of the innate immune defense barrier of the human body (Gong et al., 2009). During tissue injury, HMGB1 is secreted by activated immune cells or passively released into the extracellular environment by dying or injured cells (Dai et al., 2018). It is a pocket-sized protein, comprising 215 amino acid residues. Structurally, the protein is divided into three areas: two tandem high mobility group box domains (A and B) isolated by a short flexible linker, and a 30 amino acid, acidic C-terminal tail (Sims et al., 2010). It binds to DNA to alter the physical structure of chromatin while simultaneously maintaining genome stability and has roles in DNA processing and repair, deficient mitochondria autophagy clearance and autophagy control (Andersson and Tracey, 2011). Apart from its role in sensing and coordinating the cellular stress response inside the cell, on its secretion it also has cytokine, chemokine, and growth factor vitality, collaboratively coordinating the inflammatory and immune response with other factors as a prototypic damage-associated molecular pattern molecule (DAMP). HMGB1 activity is regulated by acetylation/deacetylation and methylation and its expression is promoted by serine phosphorylation (Bonaldi et al., 2003; Youn and Shin, 2006; Ito et al., 2007). Hyperacetylation of HMGB1 inhibits DNA binding, thus redirecting this protein to the cytoplasm for secretion (Lu et al., 2014). For example, ANG II can promote M1 macrophage polarization by upregulating the expression of HMGB1 and causing acetylation of HMGB1 and inducing HMGB1 transfer from the nucleus to cytoplasm and release by its dissociation from SIRT1 (Zhou et al., 2018). This data indicates the vital role of HMGB1 in maintaining inflammation. However, from a therapeutic perspective, silencing HMGB1 may cause the host cells to lose this protein’s key nuclear housekeeping functions. A better approach may be to improve the intracellular distribution of HMGB1 by stimulating the activity of SIRT1, thereby retaining HMGB1 in the nucleus (Le et al., 2019). This is greatly important in treating chronic diseases, especially inflammation and cancer. HMGB1 is usually actively secreted by macrophages or passively released from necrotic cells and acts as a proinflammatory mediator to induce chronic inflammation of macrophages based on the redox state causing the production of cytokines such as tumor necrosis factor alpha (TNF-α) or chemical attractants (Goto et al., 2021). Chronic inflammation can induce cancer progression. HMGB1 is secreted by cancer cells and promotes tumor growth, invasion and metastasis by binding to a variety of cell surface receptors (including receptors for advanced glycation end products (RAGE) and Toll-like receptors) (Goto et al., 2021). SIRT1 antagonizes macrophage inflammation and cancer induced by chronic inflammation by increasing SIRT1 activator NAD+ and inhibiting HMGB1 release (Yang et al., 2012). Therefore, SIRT1 and HMGB1 are believed to be important in improving chronic inflammatory degenerative diseases.

HMGB1 and SIRT1 signaling pathways are evolutionarily conserved, promoting the maintenance of homeostasis, and their interaction directly regulates inflammatory responses. Thus, this review aims to present the roles of SIRT1 and HMGB1 in inflammation, emphasizing the SIRT1-HMGB1 correlation and the resulting beneficial effects in the prevention of diseases involving inflammation.

## 2 Effect of SIRT1 on HMGB1

SIRT1 can directly inhibit the HMGB1 signal pathway to improve mammal metabolism and inflammation to maintain internal environment stability. In response to inflammatory signals, HMGB1 is hyperacetylated, resulting in its secretion from the nucleus and allowing its release from the cell. The mechanisms of signaling include lipopolysaccharide (LPS) and tumor necrosis factor-α, which promote the acetylation-dependent dissociation of HMGB1 from SIRT1, thereby indirectly increasing HMGB1 binding to the protein chromosome region maintenance 1 (CRM1), resulting in HMGB1 translocation (Mai et al., 2005; Bernier et al., 2011; Kemper et al., 2013). Substrates availability, post-translational modifications and interaction with other proteins along with changes in its expression levels co-regulate the activity of SIRT1 (Jiang W. et al., 2018; Jalgaonkar et al., 2022). SIRT1 then physically interacts with multiple lysine residues of HMGB1 at the nuclear localization signal (NLS) site through its N-terminal lysine residues to deacetylate HMGB1, resulting in HMGB1 remaining in the nucleus and reducing its cytoplasmic translocation (Xu et al., 2014; Zainal et al., 2017; Wei et al., 2019).

## 3 SIRT1-HMGB1 axis in inflammation

**FIGURE 2:**
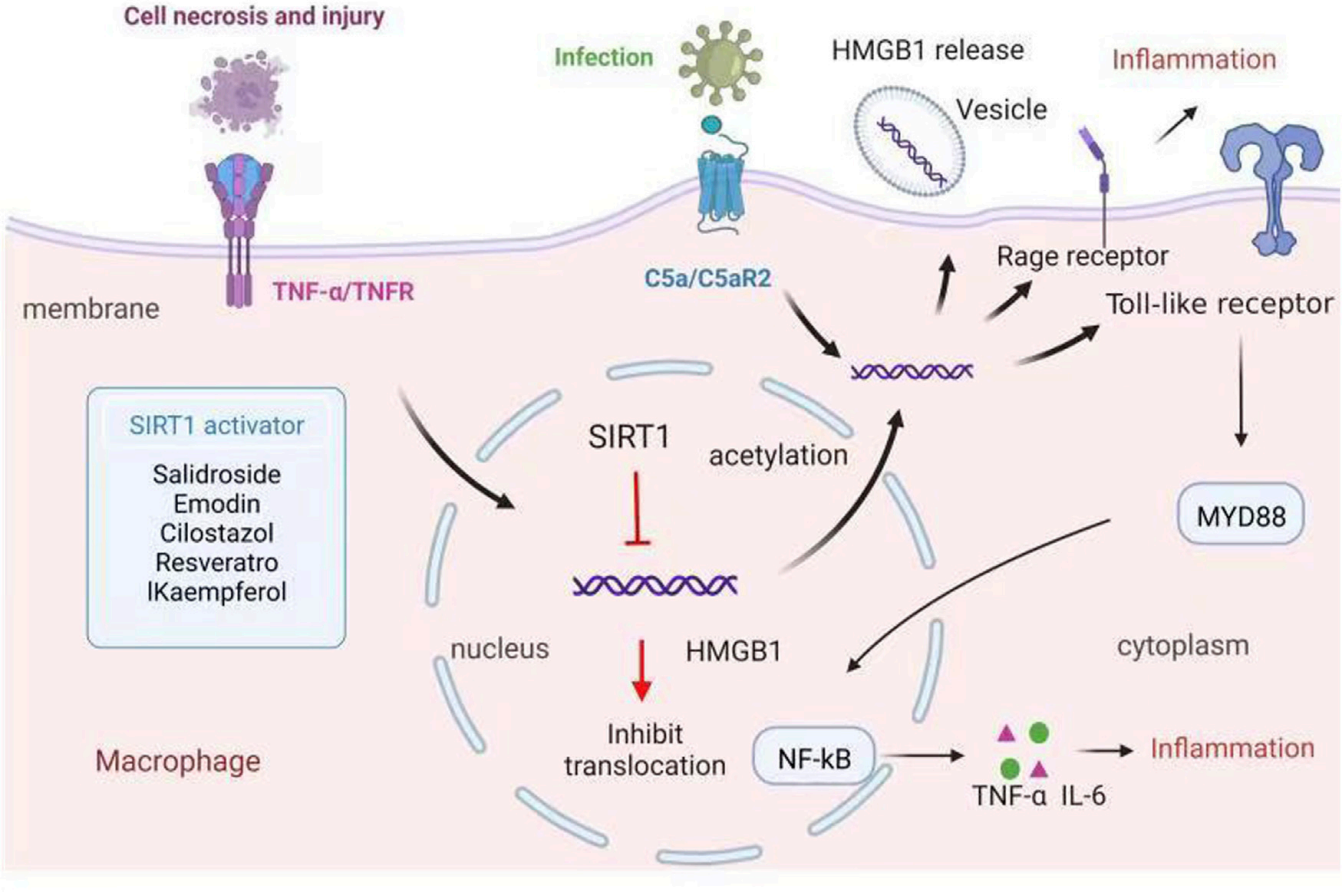
Macrophage can be influenced by surrounding necrotic cells and promote the interaction between HMGB1 and its receptor RAGE mediated by TNF-α, releasing acetylated HMGB1 and enhancing inflammatory response. When macrophage is infected, C5a binding with its receptor C5aR2 induces upregulation of HMGB1 expression in cytosyl and transfer of HMGB1 from cytosyl to the cell membrane in the vesicle. HMGB1 in the cytoplasm can activate MyD88 through TLR and then activate downstream transcription factor NF-κB, make it transfer to the nucleus, and finally promote the release of TNF-α, IL-6 and other inflammatory factors. HMGB1, as a substrate of SIRT1, can be inhibited the release under the SIRT1 deacetylation, then improving inflammation. Thus, SIRT1 activators can be used as potential agents to control inflammation by increasing SIRT1 expression. HMGB1, High mobility group box 1; RAGE, the receptor for advanced glycation end-products; MyD88, myeloid differentiation factor 88; TLR, toll-like receptor; TNF-α, tumor necrosis factor alpha; IL-6, interleukin-6.

Many studies have shown that SIRT1 is a key to reducing the translocation of HMGB1 in inflammatory responses, thus improving inflammation. Therefore, in recent years, numerous activators of SIRT1 have been investigated as potential treatments and preventative agents for inflammation-related diseases. Candidate therapeutic agents that act *via* this mechanism are reviewed in this section, organized by the type of inflammation and by disease (Figure 2; Table 1).

**TABLE 1:** Summary of direct and indirect SIRTI activators with therapeutic potential in inflammatory diseases.

Therapy	Cell types	Inhibition of HMGB1	Activated transcription factor	Main outcome	Ref.
ω-3 PUFA	Microglia	Acetylation	SIRT1	Produce neuroprotective effects for experimental traumatic brain injury	Chen et al. (2017), Chen et al. (2018a), Chen et al. (2018b)
Oleanolic acid	Brain injury cells in rats	Transfer	SIRT1	Play an anti-inflammatory role to alleviate early brain injury after subarachnoid haemorrhage	Han et al. (2021)
Baicalin	Microglia	Release	SIRT1	Reduce microglia-associated neuro inflammation and improved cute neurocognitive deficits in LPS-induced mice	Li et al. (2020)
Aloin	Human umbilical vein endothelial cells	Release	SIRT1	Treat severe vascular inflammatory diseases like sepsis and septic shock	Yang et al. (2019)
Kaempferol	Lung suffered I/R	Release	SIRT1	Improve the lung pathological injury and inhibit the release of inflammatory factors	Yang et al. (2020a)
Chrysophanol	Heart tissue	Activation	SIRT1	Attenuate DM-induced heart damage and inflammation of the heart	Xue et al. (2019)
Polydatin	HK-2 cells	Release	SIRT1	Attenuate rats’ renal tubular epithelial cell apoptosis, protect renal function and prolong survival in haemorrhagic shock rats	Xu et al. (2019b)
Salvianolic acid B	Hepatocytes	Release	SIRT1	Attenuate HFD-induced	Meng et al. (2020)
				Liver damage, hepatic steatosis, and inflammation	
PKA	Retinal endothelial Cells	Translocation	SIRT1	Reduce HMGB1-induced retinal inflammation	Liu et al. (2018)
Resveratrol	Microglia	Release	SIRT1	Exert neuroprotective effects by inhibiting the inflammatory response of microglia	Le et al. (2019)
	Murine macrophage-like RAW264.7 cells with LPS	Expression	SIRT1	Antagonize the inflammatory effects of LPS for anti-inflammation effects	Yu et al. (2019), Nebbioso et al. (2020)
Salidroside	LPS-treated macrophages	Translocation	SIRT1	Protect against sepsis-induced acute lung injury and mortality	Lan et al. (2017)
Cilostazol	RA fibroblasts	Expression	SIRT1	Inhibit HMVECs tube formation	Kim et al. (2014)
Epac1	Retinal endothelial cells	Acetylation	SIRT1	Reduce key inflammatory cascades in the retina	Jiang et al. (2018d)
Emodin	Murine alveolar epithelial cell	Expression	SIRT1	Alleviate sepsis-induced lung injury	Liu et al. (2022)
Melatonin	Murine BV2 microglia cell	Release	SIRT1	Ameliorate lipopolysaccharide-induced microglial inflammation	Chibaatar et al. (2021)
Oleanolic acid	Aneurysmal subarachnoid haemorrhage rat model	Translocation	SIRT1	Alleviate early brain injury after subarachnoid haemorrhage	Han et al. (2021)

*ω*-3 PUFA, omega-3 polyunsaturated fatty acids; LPS, lipopolysaccharide; I/R, ischemia/reperfusion; DM, diabetes mellitus; HK-2, human proximal tubular epithelial-2; HFD, high-fat diet; PKA, protein kinase A; RA, rheumatoid arthritis; HMVECs, human microvascular endothelial cells; EPAC1, exchange protein activated by cAMP 1.

### 3.1 SIRT1-HMGB1 axis in neuroinflammation

Neuroinflammation is a term used to describe the common immune responses of the central nervous system, which is the key to the pathological process of several acute and chronic brain diseases including delirium and Alzheimer’s disease (Lyman et al., 2014), primarily concerning the cells such as microglia and astrocytes. Protein tyrosine phosphatase 1B (PTP1B), as a regulator of activation of hypothalamic microglia, can enhance the LPS-induced neuroinflammatory response of microglia (Lee et al., 2020). Neuroinflammation is activated in response to central nervous system injury to initiate repair mechanisms acutely. Although acute neuroinflammation is protective, chronic neuroinflammation is often considered harmful and damaging to nerve tissue. That is, chronic neuroinflammation can aggravate, spread, and prolong central nervous system injury (Corwin et al., 2018).

#### 3.1.1 SIRT1-HMGB1 axis in traumatic brain injury

Traumatic brain injury (TBI) can be defined as the retardation of brain function, or other brain pathology diseases, triggered by external physical forces (Khellaf et al., 2019), resulting in death and disability in people with physical trauma (Vella et al., 2017), and presenting in various forms ranging from mild alterations of consciousness to a persistent comatose state and death. In the most severe form of TBI, the whole brain is affected by diffuse injury and swelling (Galgano et al., 2017). Neuroinflammation is an axiomatic physiological response to TBI. Similarly, neuroinflammation is the major cause of disability and death after TBI (Morganti et al., 2016; Tang et al., 2016). It has been shown to play a part in inducing secondary damage following brain injury that contributes to chronic neurodegeneration and neurological impairments associated with TBI (Kumar and Loane, 2012). Experimental models of TBI upon post-traumatic neuroinflammatory inhibition have been reported to improve neurological parameters (Paudel et al., 2020).

HMGB1 is among the first pro-inflammatory cytokines released following TBI, and has an initiating role in neuroinflammation, acting as the “master-switch” (Manivannan et al., 2021). Studies have shown that nucleocytoplasmic translocation and extracellular secretion of HMGB1 are increased after TBI, which activates the HMGB1/nuclear factor kappa B (NF-κB)/P65 pathway and promotes the expression of pro-inflammatory factors (Chen et al., 2018a). Omega 3 polyunsaturated fatty acids (ω-3 PUFAs) have been shown to have neuroprotective properties that regulate signal transduction in brain cells, including monoamine regulation, and are involved in the modification of receptor properties or activation of receptor signal transduction (Agostoni et al., 2017). Chen et al. (2018a) demonstrated that the treatment with ω-3 PUFAs increased SIRT1 activity following TBI, thus inducing SIRT1 to interact directly with HMGB1 and inhibiting HMGB1 acetylation. These interactions were shown to inhibit HMGB1 nucleocytoplasmic translocation and extracellular release, preventing HMGB1-mediated activation of the NF-κB signaling pathway after TBI-induced microglial activating and thus suppressing the subsequent inflammation. Therefore, it can inhibit TBI-induced inflammation, and this inhibitory mechanism may be associated with microglial activation, resulting in neuroprotective effects. Overall, the anti-inflammatory and antioxidant effects of ω-3 PUFAs demonstrated in this study show promise for the treatment of neuroinflammation.

#### 3.1.2 SIRT1-HMGB1 axis in cerebral ischemia

Cerebral ischemia occurs when a blood vessel is blocked by a thrombus or embolus [2], resulting in brain tissue damage, including cerebral infarction and neuronal cell death, which manifests clinically as ischemic stroke (Shin et al., 2020). Its result depends on how many neurons die from hypoxia in the ischemic area. After cerebral ischemia leads to brain damage and neuronal loss, it can also lead to neuroinflammation that lasts for months after stroke (Ahmed et al., 2016; Vay et al., 2018). Neuroinflammation is one of the major aspects of cerebral ischemia, and can adversely affect neurogenesis. Microglia, as the resident innate immune cells of the brain, is in charge of eliciting early and pronounced inflammatory response in the immature brain after hypoxic-ischemic (HI) insult, rather than infiltrating blood-derived macrophages (Kaur and Ling, 2009; Shankaran, 2012; Umekawa et al., 2015). Paradol, as a biotransforming metabolite of shogaol, can significantly reduce microglial activation, TNF-α and nitric oxide synthase (iNOS) expression, improve neuroinflammation and central nervous system disorders, achieving the purpose of treating cerebral ischemia (Subedi et al., 2021).

HMGB1 influences neuroinflammatory responses to cerebral ischemic injury, which conduces to the pathogenesis of cerebral ischemia. It has been suggested that HMGB1 may work as a pro-inflammatory molecule, particularly through alarmin-driven inflammatory feedback mechanisms, further exacerbating the harm during cerebral ischemic injury (Singh et al., 2016). HI, insult induces reactivity in microglia, which actively release acetylated HMGB1 (Ac–HMGB1); this, in turn, motivates the TLR4/myeloid differentiation factor 88 (MyD88)/NF-κB signaling pathway in microglia, resulting in glial–neuronal neuroinflammation, consisting of the production of a mass of pro-inflammatory molecules and mediators including interleukin (IL)-1β, IL-6, and TNF-α. It has been suggested that the expression level of SIRT1 is declined in neonatal Hypoxic-ischemic brain injury (HIBI) (Carloni et al., 2017). SIRT1 is of great value for cognitive function, neuronal plasticity, and prevention of aging-related neuronal degeneration and cognitive decline (Ng et al., 2015). A key role for SIRT1 in neuroprotection against cerebral ischemia, *via* the deacetylation and inhibition of p53 and NF-κB-induced inflammatory reaction and apoptosis, has also been indicated (Hernández-Jiménez et al., 2013). Studies have shown that resveratrol (RES) improves neurological function and neuronal damage, inhibits inflammation and neuronal apoptosis, and protects neurons from cerebral ischemia (Park et al., 2019). Animal experiments have also shown that RES can play neuroprotective and antioxidant effects on apoptosis induced by cerebral ischemia rats by increasing SIRT1 expression (Meng et al., 2015). Upregulation of SIRT1 decreases the acetylation status of HMGB1, which in turn plays a key role in the cellular response to inflammation by deacetylation-mediated HMGB1 release (Lan et al., 2017). RES treatment directly increases the interaction between SIRT1 and HMGB1 by promoting the expression and activity of SIRT1, inhibiting acetylation of HMGB1, restraining the nucleocytoplasmic translocation and subsequent secretion of HMGB1 from microglial cells, and finally weakening the downstream inflammatory cascade such as TLR4 signaling pathway caused by this molecule, ameliorating brain injury and behavioral impairment attributing to HI insult (Le et al., 2019). Therefore, RES could be investigated as adjunctive therapy for neuroinflammation; however, further clinical research for this is required.

### 3.2 SIRT1-HMGB1 axis in lung inflammation

Lung ischemia-reperfusion injury (LIRI) is a complex, inflammatory condition of the lung in the absence of infection, which involves rapid oxidative stress and subsequent responses by all cells in the lung. It ultimately leads to the breakdown of endothelial and epithelial barriers, resulting in life-threatening edema and defective gas exchange, which poses a huge threat to graft and recipient survival, resulting in ascending morbidity and mortality among lung transplant patients (Trulock et al., 2006). Innate immune cells are rapidly activated upon reperfusion, resulting in neutrophil influx, pulmonary edema and succeeding lung dysfunction (Sharma et al., 2007) *via* the production of pro-inflammatory cytokines and the activation of intricate inflammatory signaling pathways. Severe cases can cause direct tissue injury and augment pulmonary inflammation (Laubach and Sharma, 2016).

Total and extranuclear HMGB1 levels were both greatly increased after LIRI, causing lung injury in rats (Yang et al., 2020a). At the same time, HMGB1 expression can be increased in a variety of lung diseases, such as pneumonia (Ding et al., 2018), pulmonary fibrosis (Zhao et al., 2017), and chronic obstructive pulmonary disease (Jiang et al., 2019). This suggests that excessive HMGB1 may produce tissue injury and organ dysfunction in the pathogenesis of many illnesses, with either sterile or infectious origins (Yang and Tracey, 2010). Extracellular HMGB1 is involved in promoting inflammation and oxidative stress by interacting with Toll receptors and RAGE to activate NF-κB (Bortolotto and Grilli, 2017). SIRT1-related pathways are also the core components of redox signaling pathways. The ability of SIRT1 to resist oxidative stress *in vivo* was first reported by Alcendor et al. (2007). Therefore, SIRT1 can inhibit HMGB1 expression by protecting cells from oxidative stress. Yang et al. (2020a) illuminated that dietary flavonoid kaempferol significantly upregulates SIRT1 expression in rat lungs of ischemia/reperfusion (I/R), thereby reducing the expression level of HMGB1, inhibiting the release of inflammatory factors, decreasing the activity of NF-κB pathway, reducing malondialdehyde and superoxide dismutase levels, and improving pathological injury. Therefore, kaempferol may protect against LIRI and act as an anti-inflammatory and anti-oxidative stress agent *via* the SIRT1/HMGB1 axis. Kaempferol may therefore be a useful therapeutic candidate in inflammation and infection of the lung.

### 3.3 SIRT1-HMGB1 axis in arthritis

Arthritis is defined as inflammation of joints causing pain, swelling, and stiffness (Shrivastava and Pandey, 2013), used to be thought of as a life-long illness. Current treatments for arthritis have focused on disease control, and the cure still seems unreachable. Therefore, life-long therapy is required to inhibit the inflammatory process to effectively control further cartilage and bone damage (Zaninelli et al., 2021). Arthritis is the leading cause of disability in the United States (US) and other populations (Basu et al., 2018). Although the etiology and underlying mechanisms of arthritic conditions are complex, evidence suggests that the progression of this condition may be primarily driven by an increase in oxidative stress and inflammation (Geyer and Schönfeld, 2018). Among these conditions, rheumatoid arthritis (RA) has the characteristic of uncontrolled proliferation of synovitis, and inflammatory synovitis, accompanied by neutrophil infiltration, fibroblast proliferation, and angiogenesis in patients with RA. Uncontrolled proliferation of the synovial lining results in microenvironmental variations, leading to the chronic production of low oxygen tension and pro-inflammatory cytokines. The normal cellular response to hypoxia is mediated by hypoxia response genes, including hypoxia-inducible factor (HIF), such as HIF-1α and HIF-2α (Semenza, 2007). The HIF-1 signaling pathway is activated under hypoxia condition and subsequently induces downstream vascular endothelial growth factor (VEGF) and Notch signaling, which accelerates angiogenesis of articular cartilage. Therefore, it is a target for anti-angiogenic therapy in RA (Chen et al., 2019).

Extracellular HMGB1 takes a part of a coupling factor between hypoxia and inflammation in arthritis and localizes preferentially to regions of tissue hypoxia in arthritic lesions (Hamada et al., 2008). HMGB1 can be actively released by macrophages and passively secreted from necrotic cells. That is, molecules derived from exogenous pathogens stimulate the innate immune system to promote the active release of HMGB1. In the absence of invasion, ischemiaor cellular injury and hypoxia can motivate the passive release of HMGB1 (Andersson and Tracey, 2011), increasing extracellular HMGB1. Kim et al. (2014) confirmed that the signaling pathways associated with HMGB1-induced downstream molecule HIF-1 expression and VEGF release involve NF-κB activation in RA synovial fibroblasts (SF), which results in the disruption of bone deformation, articular cartilage and synovial proliferation, aggravating the pathogenesis in RA disease (Kaur et al., 2020). SIRT1 expression was found to be significantly decreased between 3 and 24 h after exposure to HMGB1 (Pullerits et al., 2003). SIRT1-induced deacetylation is involved in restraining HIF1 signaling. Cilostazol is a vasodilating antiplatelet drug, the effects of cilostazol mainly help to stimulate NO production, inhibit platelet aggregation, vasodilatation and enhance peripheral blood flow (Elrashidy and Hasan, 2021). Cilostazol can activate SIRT1, and therefore, induces dual effects in RA SFs: on one hand, it increases HIF-1a deacetylation by enhancing SIRT1 activity, thus blocking VEGF expression and leading to the suppression of synovial angiogenesis; on the other, it inhibits HMGB1 expression, thereby suppressing HIF-1a and VEGF expression. It has therefore been shown to have anti-angiogenic effects *in vitro* and in a collagen-induced arthritis (CIA) mouse model. Cilostazol can be a potential candidate drug for preventing and treating arthritis.

### 3.4 SIRT1-HMGB1 axis in hepatocyte inflammation

Non-alcoholic fatty liver disease (NAFLD) is the main cause of chronic liver disease and has become an increasingly serious public health problem on a global scale (Tarquini et al., 2010). Inflammatory reaction plays a key role in this disease. One-quarter of NAFLD patients are judged with non-alcoholic steatohepatitis (NASH), where histological evidence shows not only the fatty accumulation of liver cells but also hepatocyte damage and death because of the long-term inflammatory response (Zhu et al., 2021). Both hepatocyte injury and liver inflammation are implicated in the pathogenesis of NASH, as damaged liver cells release inflammatory factors that induce inflammation, and as a result that downward spiral as inflammation further causes hepatocyte damage (Zhu et al., 2021). Abnormal immune responses and immune cell infiltration caused by various liver injuries (such as viral or parasitic infection, drug toxicity, alcoholism and metabolic diseases) can disrupt the immune state of the liver and lead to liver inflammation. Mesenchymal stem cells (MSCs) are multipotent progenitor cells that can differentiate into osteoblasts, adipocytes and chondrocytes, and have unique immunomodulatory effects on numerous effector immune cells such as T lymphocytes, B cells and natural killer cells (Liu et al., 2021a). MSCs are usually provided by isolating them and maintaining them in human liver tissue culture, called liver MSCs (Kholodenko et al., 2019). Preclinical and clinical studies have shown that MSC transplantation can reduce liver inflammation and thereby improve liver cell regeneration, which can help patients with liver injury through an immune-mediated pathway (de Miguel et al., 2019). Yang et al. (2021) also indicated that Mesenchymal stem cells-conditioned medium (MSC-CM) enhanced the biological functions of mitochondria, inhibited inflammation, and prevented cell apoptosis both *in vivo* and *in vitro*, which significantly improved NAFLD. These positive effects were closely related to the upregulation of SIRT1. SIRT1 has been reported to have anti-steatosis and anti-inflammatory movements in the pathogenesis of NAFLD (Meng et al., 2020). Therefore, these results suggest that SIRT1 may be closely related to the mechanism controlling hepatic steatosis and inflammation in NASH.

HMGB1, an inflammatory mediator, secreted by damaged liver cells, prolongs the inflammatory response, playing a key role in diverse pathogenic mechanisms in liver disease, such as inflammation, fibrosis, steatosis and tumorigenesis (Li et al., 2011; Gan et al., 2014; Vicentino et al., 2018). Liu et al. found that the translocation of HMGB1 from the cytoplasm to the nucleus increased in acute liver failure (Wang et al., 2008; Liu and Yao, 2010); overexpression of HMGB1 in hepatocytes also particularly enhanced the risk of liver injury when sepsis happened (Xu et al., 2014). Recent studies have reported that HMGB1 levels are enhanced in NAFLD in both animal models and a clinical setting in humans and that inhibiting HMGB1 leads to a remarkable decrease in inflammatory responses in NAFLD (Li et al., 2011; Montes et al., 2015). Also, Rauh et al. (2013) indicated that HMGB1 was one of the main SIRT1 substrate candidates. Rabadi et al. (2015) have also demonstrated that the inflammation-induced suppression of SIRT1 inhibits HMGB1 deacetylation and promotes its nuclear-to-cytoplasmic translocation and systemic secretion, therefore keeping inflammation. Meng et al. (2020) indicate that salvianolic acid B (SalB), can suppress the relocation and secretion of HMGB1 by upregulating SIRT1 in the liver parenchymal cells during NAFLD. Meanwhile, SIRT1-mediated deacetylation can result in the resveratrol-mediated suppression of HMGB1 nuclear-to-cytoplasmic translation in sepsis-induced liver injury (Xu et al., 2014). Therefore, the anti-inflammatory SIRT1/HMGB1 pathway may act as a typical pharmacological target to attenuate the progression of NAFLD for the development of new drugs.

### 3.5 SIRT1-HMGB1 axis in inflammatory responses during pregnancy

Preeclampsia (PE), a pregnancy-specific disorder linked to inadequate maternal inflammation, oxidative stress, placental ischemia, vascular endothelial cell dysfunction and injury of the blood vessel (Feng et al., 2016a), can result in high maternal and perinatal morbidity and mortality (Feng et al., 2016b). Pregnant woman with PE is prone to autoimmune diseases, maternal renal disease and metabolic syndromes. This aberrant inflammatory activation can also result in harmful pregnancy outcomes, such as preterm birth or miscarriage (Nadeau-Vallée et al., 2016). Recently, a growing number of researches suggest that PE possibly originates from poor placental development. The placenta protects the fetus from maternal immune responses owing to its biological barrier between the mother and the fetus. It has been believed that HMGB1 levels are enhanced in the syncytiotrophoblast of the placenta, and in the serum in both severe PE and early onset PE, the expression of serum cytokine and chemokine levels is also significantly elevated (Chen et al., 2016). This indicates that HMGB1 plays a crucial pathogenic role in PE. In this condition, excessive inflammation and oxidative stress can give rise to the dysfunction of vascular endothelial cells, triggering their death. Necrotic cells release HMGB1, further contributing to the inflammation, in a positive feedback loop (Yin et al., 2017). Yin et al. (2017) found that SIRT1 can inhibit HMGB1 release in cell models of PE and further suppress the pro-inflammatory effects of HMGB1. Increasing SIRT1 levels has been shown to improve inflammatory and stress responses and prevent vascular endothelial cells from death. Therefore, placental SIRT1 is likely protective against PE. The SIRT1/HMGB1 pathway may therefore be a potential therapeutic target for alleviating inappropriate inflammatory responses during pregnancy.

### 3.6 SIRT1-HMGB1 axis in sepsis

Sepsis, infected with systemic signs of infection, is characterized as a systemic inflammatory response syndrome (Galtrey et al., 2015). The characteristics of sepsis are physiologic, biochemical and pathologic abnormalities induced by infection, also include dysfunctional blood coagulation, dysregulated inflammation, and multiple organ damage, and severe sepsis is characterized by sepsis plus sepsis-induced organ dysfunction or tissue hypoperfusion (Lan et al., 2017). The morbidity and mortality related to severe sepsis are enhanced and sepsis can lead to death in intensive care (Gentile and Moldawer, 2014). Sepsis has a biphasic inflammatory process: producing pro-inflammatory cytokines in the early phase, including TNF and interleukins, and the late phase is mediated by HMGB1 (Abraham et al., 2000). HMGB1 rearranges the actin cytoskeleton into a contractile phenotype through the downstream effector of late action, disrupting the endothelial cell barrier and increased mortality in sepsis (Galtrey et al., 2015). In addition, HMGB1 expression was found to be increased in septic acute kidney injury (AKI) mouse models, and serum HMGB1 levels were positively correlated with the severity of sepsis (Xu et al., 2020). Therefore, therapeutics targeting this protein may be a new method for targeting persistent inflammation in people with sepsis.

Extracellular HMGB1, a mediator of late sepsis, acts as a major mediator in both acute and chronic inflammation (Abraham et al., 2000). Nuclear-to-cytoplasmic HMGB1 translocation is increased in acute lung injury, and in this context, inhibiting HMGB1 secretion improves sepsis-induced organ injury and systemic inflammatory response syndrome (Wang et al., 2009). Accordingly, inhibition of HMGB1 translocation and/or secretion therapeutically may have a protective effect on acute lung injury induced by sepsis. Wei et al. (2019) found that SIRT1 activation resulted in deacetylation of HMGB1 and attenuation of its nuclear-to-cytoplasmic translocation, both *in vivo* and *in vitro* in a model of sepsis-associated AKI. Besides, the deacetylation of HMGB1 mediated by SIRT1 inhibited inflammation, attenuated renal function, and crucially lengthened survival time in septic mice. Activated SIRT1 has been shown to directly interact with HMGB1 by its NH_2_-terminal lysine residues 28–30, and then suppressing the secretion of HMGB1 and improving survival time in an experimental model of sepsis (Wei et al., 2019). On the contrary, inflammatory stimulation accelerates the acetylation of HMGB1 and promotes its secretion by eliciting its separation from SIRT1 (Wei et al., 2019). Lan et al. (2017) indicated that salidroside was found to prohibit the expression of pro-inflammatory cytokines (TNF-α and IL-6) *via* SIRT1-mediated suppression of the NF-κB activation pathway in the early septic phase. In the late septic phase, salidroside also prevented acute lung injury *via* the SIRT1-mediated HMGB1 nucleocytoplasmic translocation pathway induced by sepsis. Salidroside has a bipartite curative effect, ameliorating both early and late phase inflammation associated with sepsis. Therefore, salidroside is expected as a therapeutic agent in a septic mouse model. Overall, this research demonstrates that salidroside may play a protective therapeutic role in attenuating the progression of sepsis *via* the anti-inflammatory SIRT1/HMGB1 pathway.

### 3.7 SIRT1-HMGB1 axis in diabetes-related inflammation

Diabetes, a metabolic disease caused by defects in insulin secretion and/or action and characterized by chronic hyperglycemia, can facilitate atherosclerotic illness and improves the brain, heart and lower limb arteries (Mäkimattila et al., 1996). It has a series of features including hyperglycemia, insulin resistance, insulin deficiency, and varied pathologies in many organs, such as the liver, the nerves, and the glomeruli in the kidneys. The importance of the role of inflammation in diabetes and diabetic complications, particularly in the retina and myocardial injury, is becoming increasingly evident. Increasing patients and the defect of pretreatments with this condition demonstrates a pressing requirement for the progress of novel targeted agents for metabolic pathways resulting in diabetes and diabetic complications. Many signaling cascades have been shown to play a role in this condition; however, no therapeutic agents currently exist. Therefore, more additional targeted treatment still needs to be identified. Some research has indicated that the SIRT1-HMGB1 axis can play a major pathway for novel beneficial adjustment.

#### 3.7.1 SIRT1-HMGB1 axis in retinal complications

Diabetic retinopathy (DR), as a frequent complication especially in type 2 diabetes, has been more and more associated with inflammation (Zhang et al., 2012). It is characterized by abnormal retinal neovascularization, endothelial dysfunction and vascular inflammation (Robinson et al., 2020). It has been reported that insulin-like growth factor binding protein 3 (IGFBP3) is neuroprotective in the retina, reducing retinal inflammation induced by injury (Jiang Y. et al., 2018). It has also been shown that IGFBP3 reduces hepatic inflammatory response *via* the reduction in the activity of NF-κB and Janus kinase (JNK). Simultaneously, exchange protein activated by cAMP 1 (EPAC1), a guanine nucleotide exchange factor, can prohibit inflammatory pathways, block retinal leukostasis, and decrease entire HMGB1 levels in retinal capillary endothelial cell (REC) (Jiang et al., 2017). HMGB1 can directly bring about apoptosis in REC and diabetic human and rat retinas (Mohammad et al., 2015). Lossing EPAC1 statistically greatly cuts down IGFBP3 levels in the retinal vasculature in the mouse. EPAC1 activates IGFBP3 to raise SIRT1, reducing the acetylation of HMGB1. High glucose levels were also shown to increase the acetylation of HMGB1 in RECs, and this effect was inhibited by the EPAC1 agonist (Jiang Y. et al., 2018). Protein kinase A (PKA) can also mediate reductions in cytoplasmic HMGB1 by increasing the activity of both IGFBP3 and SIRT1, which has a protective effect on the DR. Thus, SIRT1 and HMGB1 axis is a promising pathway for the development of a therapeutic intervention for vascular inflammation. Simultaneously resveratrol replenishes the expression of retinal SIRT1. Consequently, resveratrol could be considered a novel therapeutic candidate in the context of DR through the SIRT1/HMGB1 pathway.

#### 3.7.2 SIRT1-HMGB1 axis in myocardial injury

Myocardial injury is another serious complication of diabetes, leading to the death of the myocardial cell (MC) and harming the prognosis of patients. Inflammatory cytokines promote the initiation and development of heart injury. HMGB1 has been shown to participate in varied pathophysiological signaling pathways caused by the physiological environment of diabetes, and perform various functions such as activating endothelial cells, expressing chemokine receptors, producing inflammatory factors, and promoting cardiomyocyte necrosis or necroptosis (Ghigo et al., 2014). Cardiomyocytes and cardiac fibroblasts can release HMGB1, and HMGB1 can, in turn, facilitate cardiac injury or cardiac fibrosis. Reactive oxygen species (ROS) resulting from heart injury can promote HMGB1 translocation from the nucleus to the cytosol, and then boost autophagic flux, suggesting that translocation of HMGB1 induces autophagy after long-term cellular stress. In addition, HMGB1 facilitates the recruitment of inflammatory cells, including macrophages and neutrophils, to the injured heart through chemokines [e.g., chemokine ligand 12 (CXCL12)]/chemokine receptor 4 (CXCR4). After heart injury, macrophages in the cardiac resident migrate to external immune organs. Stressed cardiomyocytes or immune cells produce HMGB1 and angiotensin Ⅱ (ANG II), which can gather Ly6C + monocytes into the heart of injury and recombine the infiltrated monocytes into M1 macrophages. The recombined M1 macrophages can contribute to CD4 (+) T cell extension and cardiac injury (Lu et al., 2019). Besides, HMGB1 activates NF-κB to promote the expression of pro-inflammatory factors (Lotze and Tracey, 2005). SIRT1 reduces the production and activation of inflammatory cytokines, resulting from inhibiting NF-κB transcriptional activity through deacetylation of the p65 subunit. Xue et al. (2019) indicated as an anti-inflammatory drug, chrysophanol has a protective effect on heart damage induced by diabetes, suggesting that this drug may attenuate inflammatory responses through upregulation of SIRT1, resulting in downregulation of the HMGB1/NF-κB pathway. All this evidence indicates that chrysophanol can be a useful agent for the treatment of myocardial illness resulting from diabetes.

### 3.8 SIRT1 in HMGB1-mediated tumor occurrence

Globally, cancer incidence and mortality are on the rise (Xu et al., 2019a). Cancer is one of the principal causes of death worldwide in countries of all income levels (Jemal et al., 2011). In the United States, up to 25% of deaths in humans presently are distinctly related to cancer (Balachandran and Govindarajan, 2005). Cancer is typically treated with a combination of surgery, chemotherapy, ionizing radiation therapy, hormonal therapy, and targeted therapy. Recently, there have also been profound breakthroughs in cancer treatment including checkpoint blockade immunotherapies (Siegel et al., 2020). Though there have been many recent therapeutic advances in cancer treatment, it is still considered an incurable disease in many instances (Wang et al., 2018). Thus, more effective or adjunctive therapies are needed to prevent and treat these currently intractable cancers. Some potential anticancer drugs that act *via* the HMGB1 and SITR1 pathways have been identified. The following sections provide an overview of these drugs (Table 2).

**TABLE 2:** Summary of direct and indirect SIRTI activators with therapeutic potential in tumor occurrence.

Therapy	Cell types	Inhibition of HMGB1	Activated transcription factors	Main outcome	Ref.
Resveratrol	Liver cell	Overexpression and hyperacetylation	SIRT1	Reduce liver damage after liver resection	Yu et al. (2019)
Doxorubicin	Ovarian cancer cell	Expression or acetylation	SIRT1	Suppress migration, invasion or angiogenesis of ovarian cancer cells	Zhu et al. (2017), Jiang W. et al. (2018)
Emodin	Osteosarcoma cell	Acetylation	SIRT1	Alleviate tumour angiogenesis	Qu et al. (2015)

Traditional anti-inflammatory drugs have anti-cancer and anti-tumor properties. For example, numerous epidemiological studies have revealed that malignancy and cancer incidence are credibly reduced after using nonsteroidal anti-inflammatory drugs (NSAID) for a long time, including aspirin, celecoxib, diclofenac, diflunisal, sulindac, and tolmetin (Khandia and Munjal, 2020). So cancer is closely related to inflammation. Chronic inflammation is involved in all stages of cancer development and the inflammatory tumor microenvironment is a hallmark of cancer. According to epidemiological and clinical estimation, about 25% of cancers are associated with chronic and acute inflammation (Wu et al., 2017). Therefore, our understanding of the pathological mechanisms of cancer can contribute to recognizing the link between cancer and inflammation. In 1863, Virchow hypothesized that the sites of cancer occurrence are triggered by chronic inflammation and that some classes of irritants enhance cell proliferation by eliciting tissue injury and inflammation (Balkwill and Mantovani, 2001). Indeed, it is now clear that sustained cell proliferation in an environment rich in inflammatory cells, growth factors, activated stroma, and DNA damage-promoting agents promotes and/or maintains neoplastic risk (Singh et al., 2019). The pro-tumor activity of inflammatory cells includes stimulating growth and survival factors secretion, spurring DNA damage, motivating angiogenesis and lymphangiogenesis, remodeling the extracellular matrix to facilitate invasion, disseminating tumor cells *via* lymphatic vessels and capillaries, and avoiding host defense mechanisms (Singh et al., 2019). Susceptibility to cancer and subsequent disease severity may be associated with functional polymorphisms of inflammatory cytokine genes; experimental genetic deletion or inhibition of these cytokines inhibits the development of cancer (Balkwill and Mantovani, 2001). Anti-inflammatory therapy is effective during the early neoplastic progression and malignant conversion stages of cancer development. For instance, ulcerative colitis and Crohn’s disease can increase the neoplastic risk and this process is, reduced by the use of anti-inflammatory agents for colitis (Zappavigna et al., 2020). Also, the use of NSAIDs decreases the number and size of colonic polypsin in patients with familial adenomatous polyposis; similarly, aspirin has also been found to confer a protective effect on the colorectum in patients with Lynch syndrome (Park et al., 2014). Therefore, anti-inflammatory therapy may be a useful option for treating early-stage diseases and precancers.

#### 3.8.1 SIRT1 in HMGB1-mediated hepatocellular carcinoma

Hepatocellular carcinoma (HCC), or primary liver cancer, is one of the major causes of death related to cancer worldwide (Gravitz, 2014). Although great progress has been made in the diagnosis and treatment, the treatment difficulty and morbidity of liver cancer are still high (Ferlay et al., 2010). Hepatectomy is the most commonly used and effective treatment for liver cancer in the clinic (Wang et al., 2013). However, this may change the structure and function of the liver, leading to liver failure (Jin et al., 2013). Accordingly, it is still important to develop new therapeutic methods to treat postoperative liver failure. HMGB1 is released by injured liver cells, which may prolong inflammatory responses and promote the progression of liver disease. SIRT1 levels in normal liver tissue are very low, but overexpression in liver cancer tissues and cell lines shows that SIRT1 has a key role in liver cancer (Portmann et al., 2013). Numerous *in vivo* and *in vitro* studies have also indicated the anti-tumor effects of resveratrol on the initiation and progression of cancer (Yu et al., 2019). Yu et al. (2019) found that HMGB1 expression and acetylation levels in a rat model of liver resection were enhanced and resveratrol could prohibit this effect. They also found that resveratrol treatment can also prohibit the downregulation of SIRT1 in liver tissues resulting from surgical resection. HMGB1, as a substrate of SIRT1, can be deacetylated by SIRT1. Therefore, RES has the potential to protect the liver from hepatectomy injury, which is closely related to the SIRT1-HMGB1 axis.

#### 3.8.2 SIRT1 in HMGB1-mediated ovarian cancer

Ovarian cancer is the second leading cause of gynecologic cancer death in women worldwide (Lheureux et al., 2019). Ovarian cancer is frequently not diagnosed until it is at an advanced stage owing to a lack of specific symptoms; late diagnosis renders it hard to treat (Stewart et al., 2019). Early studies have indicated that HGMB1 has a role in ovarian cancer pathogenesis. The expression of HMGB1 in tissues and serum of ovarian cancer patients was higher than that of benign tumor or normal control group (Wang et al., 2015). Seidu et al. (2017) showed that HMGB1 upregulation may facilitate migration and invasion in ovarian cancer. Jiang W. et al. (2018) found that the regulation of cell migration, invasion and angiogenesis by HMGB1 was governed by SIRT1. SIRT1 can improve the prognosis of ovarian cancer. Ding et al. proved that alisertib could inhibit epithelial-mesenchymal transformation and induce autophagy in ovarian cancer cells by increasing SIRT1 expression (Wang et al., 2019). Meanwhile, overexpression of SIRT1 can effectively inhibit the expression and acetylation of HMGB1, thus inhibiting the migration, invasion and angiogenesis of ovarian cancer (Jiang W. et al., 2018). The SIRT1/HMGB1 axis may therefore be a key therapeutic target for inhibiting ovarian cancer migration, thus attenuating the progression of this disease.

#### 3.8.3 SIRT1 in HMGB1-mediated osteosarcoma

Osteosarcoma (OS) originates from MSCs and is considered the most frequent malignant bone tumor (Liu et al., 2021b). It is characterized by osteoid tissue generation or immature bone formation, especially in adolescents (Spraker-Perlman et al., 2019). With the introduction of neo-adjuvant chemotherapy in the 1970s, disease prognosis improved from 17% to a 5-years survival rate of 60%–70%, but there has been no significant improvement since then. There is an urgent need for new and innovative treatment strategies to supplement traditional approaches to improve the prognosis of patients with OS (Liu et al., 2021b). Except for cell proliferation and invasion, OS is also characterized by angiogenesis (Mikulić et al., 2004). Qu et al. (2015) found that in nude mice bearing human OS xenograft tumors, HMGB1 administration significantly increased angiogenesis in the tumor tissue. Recent research has also shown that angiogenesis was inhibited by SIRT1 activation, which downregulated VEGF transcription and inhibited angiogenesis induced by HMGB1 (Kim et al., 2014). Emodin, as a compound, deacetylates HMGB1 and attenuates angiogenesis induced by HMGB1 in OS by increasing SIRT1 expression and its deacetylation activity (Qu et al., 2015). Therefore, the clinical application of emodin may constitute an effective treatment strategy for OS in the future by acting on the SIRT1-HMGB1 axis.

## 4 Summary

Inflammation includes diabetes, cardiovascular diseases, eye disorders, arthritis, obesity, autoimmune diseases, and inflammatory bowel reaction (Arulselvan et al., 2016). The inflammatory process includes dilating veins and arterioles, increased vascular permeability, and blood flowing into surrounding tissues by the extravasation of leukocytes (Henson et al., 1984). SIRT1 and HMGB1 directly interact with one another, forming a stable complex in cells (Hwang et al., 2015). It is of great significance in controlling the progress of inflammation. SIRT1 generates a principal function in controlling the growth and progression of inflammation, exerting anti-inflammatory effects mainly by inhibiting the transcription of inflammation-related gene HMGB1 (Zhang et al., 2010). In the process of oxidative stress and cell necrosis, the integrity of the plasma membrane is destroyed, conducing to the interaction between HMGB1 and its receptor (notably, the RAGE) to promote the release of acetylated HMGB1 from cells, and the extracellular HMGB1 enhances inflammatory and immune responses (Hofmann et al., 1999; Park et al., 2006; Venereau et al., 2012; Yang et al., 2020b). HMGB1, as a substrate of SIRT1, is deacetylated by SIRT1 and left in the nucleus to alleviate the symptoms of inflammation significantly.

SIRT1 and HMGB1 are ancient signaling pathways that regulate metabolically and inflammation in mammals by opposite control mechanisms. In recent years, several studies have found a close relationship between the two proteins: SIRT1 can prohibit HMGB1 signaling directly, promoting its deacetylation and reducing its cytoplasmic translocation (Rabadi et al., 2015), resulting in decreased inflammatory responses (Petrovič, 2014). In turn, the HMGB1 pathway suppresses its downstream targets, which inhibits SIRT1-mediated functions. Given that SIRT1 and HMGB1 signaling pathways have antagonistic effects, these pathways can control many metabolic and inflammatory switches physiologically related to maintaining cellular and organismal homeostasis. Understanding this interaction deeply may contribute to providing brand new and valuable clinical targets for treating cancer and other conditions involving inflammation.

Some anti-inflammatory agents, including ω-3 PUFAs, oleanolic acid, baicalin, aloin, kaempferol, chrysophanol, and polydatin suppress the HMGB1/TLR4/NF-κB signaling pathway through activating SIRT1 (Matsuzawa-Ishimoto et al., 2017), and can yield improvements in neuroinflammation, arthritis, hepatocyte inflammation, and complications resulting from diabetes. Other pharmaceuticals include antitumor drugs (e.g., resveratrol, doxorubicin, and emodin), which can enhance the efficacy of treatments for certain cancers, such as hepatocellular carcinoma, ovarian cancer, and OS. These drugs increase interaction between SIRT1 and HMGB1 directly by enhancing the expression and activity of SIRT1 and reducing HMGB1 acetylation, inhibiting the nuclear-to-cytoplasmic translocation and subsequent secretion of HMGB1 from cells, and attenuating the downstream inflammatory cascade. The involvement of many molecules in this process indicates that there are multiple options for novel therapeutic approaches yet to be identified, developed, and put into clinical practice. Currently, many studies have focused on mouse models, and clinical studies in humans are required to confirm the mechanisms of action, efficacy, and safety of potential therapies. The anti-inflammatory effects caused by these molecules make their protective effect on inflammation obvious. At the same time, the field involving HMGB1 to induce SIRT1 activation has not been widely explored. Further research is needed to understand the effect of HMGB1 on SIRT1 better. Taken together, the anti-inflammatory effects induced by these molecules provide theoretical support for further exploring inflammatory pathways. SIRT1/HMGB1 pathway offers a new therapeutic target for inflammatory diseases.
